# Atypical Presentation of Testicular Adrenal Rest Tumor (TART) Leading to Bilateral Partial Orchiectomy in a 31-Year-Old Adult Revealing Primary Adrenal Insufficiency with *CYP11A1* Deficiency

**DOI:** 10.1155/2021/5889007

**Published:** 2021-12-23

**Authors:** Cyril Garcia, Marie Dusaud, Paul Chiron, Mathilde Sollier, Sika Nassouri, Lionel Groussin, Mathilde Sibony, Claire Goursaud, Florence Roucher-Boulez, Lyse Bordier

**Affiliations:** ^1^Hôpital d'Instruction des Armées BEGIN, Service d'endocrinologie, 69 avenue de Paris, Saint-Mande 94160, France; ^2^Hôpital d'Instruction des Armées BEGIN, Service d'urologie, 69 avenue de Paris, Saint-Mande 94160, France; ^3^Hôpital COCHIN, Service d'endocrinologie, 27 rue du Faubourg Saint Jacques, Paris 75014, France; ^4^Hôpital COCHIN, Service d'anatomopathologie, 27 rue du Faubourg Saint Jacques, Paris 75014, France; ^5^Hospices Civils de Lyon-Bron, Laboratoire de Biochimie et Biologie Moléculaire Grand Est, Lyon 69000, France

## Abstract

Adrenogenital syndrome is commonly associated with a deficiency in 21-hydroxylase but can be present in other rare enzymatic blocks. We report here the case of a 31-year-old man who presented with bilateral painful testicle lesions leading to bilateral partial orchiectomy as they were suspected for malignancy. These lesions were finally identified as benign testicle adrenal rest tumors (TARTs), and the patient was actually belatedly diagnosed with primary adrenal insufficiency due to 2 mutations of the *CYP11A1* gene encoding the cholesterol side-chain cleavage enzyme (P450scc); the mutations were 940G > A (p.Glu314Lys) and c.1393C > T (p.Arg465Trp). The same mutations were found in his 29-year-old sister, who was then also diagnosed for primary adrenal insufficiency. Deficiency in P450scc is an extremely rare genetic autosomal recessive disorder with around 40 described families in the literature and 30 different mutations. As the diagnosis of delayed onset of P450Scc mutation is difficult, this case illustrates the need for a systematic endocrinological assessment in any case of bilateral testicle lesions, thus avoiding unnecessary surgery.

## 1. Introduction

Adrenogenital syndrome is commonly associated with an enzymatic block and congenital adrenal hyperplasia (CAH), in which gonadal lesions develop from adrenal-like cells. In these cases, increased levels of ACTH are associated with hyperplasia of ACTH-sensitive tissues, mainly the adrenals, but also within other sites like the testis or ovaries [1]. These testicle lesions are benign and so-called TTAGS (testicular tumor of adrenogenital syndrome) or better known as TARTs (testicular adrenal rest tumors) [1, 2].

TARTs were mainly described in defects in 21-hydroxylase, which are the most frequent enzymatic blocks representing 90 to 94% of CAH [1, 3]. TARTs can be present in other blocks, such as in 17alpha hydroxylase, P450Scc, 3 beta-hydroxysteroid dehydrogenase, and 11 beta-hydroxylase deficiencies [4]. TARTs can never occur in adrenal insufficiency of autoimmune origin, despite high rates of ACTH [4].

We report here the cases of a young man and his sister, who were belatedly diagnosed with primary adrenal insufficiency due to mutations of the *CYP11A1* encoding the cholesterol side-chain cleavage enzyme (P450scc), the first rate-limiting step of steroidogenesis.

## 2. Case Report

A 31-year-old man was referred to the urologist for painful bilateral testicular lesions. No other genital lesions were observed, and in particular, no hypospadias. The patient did not report any decrease in libido or erectile dysfunction and had normal ejaculations. The blood pressure was 106/63 mmHg, the heart rate was 67 beats per minute, the height was 174 cm, the weight was 66 kg, and the BMI was 21.8 kg/m^2^. In his family, his sister's height was 1.58 m, her weight was 52 kg, and her BMI was 20.8 kg/m^2^. As his mother's height was 155 cm and his father's height was 158 cm, the target height was estimated at 163 cm ((mother + father)/2). The patient was classified as Tanner stage V because the penis was of adult size. Only facial hair was limited requiring only one shave per week. Electrolyte values were as follows: urea 1.9 mmol/L, creatinine 61 µmol/L, sodium 141 mmol/L, and potassium 4.2 mmol/L. Despite the fact that testosterone measurement was not performed, hypogonadism was suggested by bone densitometry, which showed osteopenia; the lumbar T score was -1.8 SD and the femoral T score was -1.2 SD.

**Figure 1:**
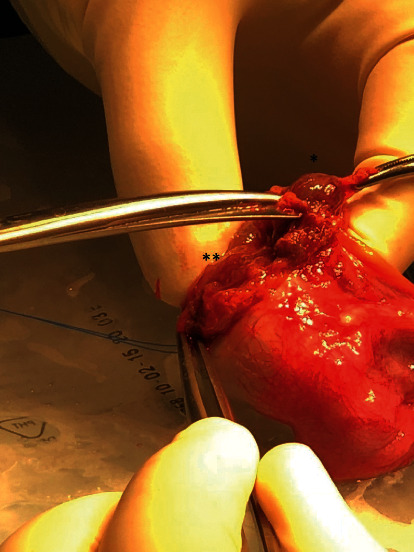
Perioperative view: partial right orchiectomy. Open testicular albuginea, tumor enucleation, and adjacent healthy parenchyma. ^*∗*^Testicular adrenal rest tumor. ^*∗∗*^ Healthy testicular pulp.

Ultrasonography revealed bilateral, heterogeneous, and vascularized lesions, localized on the top of both testicles, within the testicle hilus. Testicles were found to be hypotrophic as measured at 6 to 7 ml (normal range, 15–30 ml). The ultrasound report did not point to a benign tumor (notably, it did not formally rule out a testicular germ cell tumor). The extemporaneous analysis of the tumor suspected a Leydig cell tumor. Therefore, bilateral inguinal testicular exploration with partial orchiectomy was performed in the first instance, according to the Cancerology Committee of the French Association of Urology (CCFAU) recommendations and the European Association of Urology (EAU) recommendations. Bilateral partial orchiectomy was performed to preserve fertility (Figure 1). No preoperative hormonal analysis was made as no endocrine aetiology was evoked at that time.

The lesions were first considered as benign Leydig cell hyperplasia at anatomopathology. The tumors were measured macroscopically at 2 and 2.5 cm for the right and left lesions, respectively. They were found to be brown to orange and multimicronodular. Microscopically, the tumors were made of eosinophilic cells with regular round nuclei. In some cells, brown pigments of lipofuscin were observed. Calcification and adipose metaplasia were also observed. As the surgeon first considered these lesions as potentially malignant, classical immunohistochemistry was performed in this setting; however, these cells were first difficult to differentiate from normal Leydig cells, as immunochemistry was not specific. Lastly, because of bilateral lesions, testicular adrenal rest tumors (TARTs) were suspected, although no Reinke crystals were seen [1, 5–7].

The patient was then referred to the endocrinologist for testosterone replacement and hormonal evaluation. Presentation was marked by diffuse melanodermia and important asthenia, since adolescence.

Patient's hormone profile is presented in Table 1. Results were consistent with postoperative hypergonadotropic hypogonadism (low testosterone and elevated gonadotrophins, FSH, and LH), but primary adrenal insufficiency was also revealed (low cortisol with elevated ACTH). Antibodies against 21-hydroxylase were <0.2 mUI/L. Adrenal androgens and most of the steroids of the adrenal cortex were low (Table 1). Atrophic adrenal glands were observed on computed tomography. As the patient was then explained that a genetic aetiology was suspected, he asked for his 29-year-old sister to also be screened for adrenal insufficiency as she has suffered from unexplored asthenia since childhood. This young woman also presented melanodermia. The first menstruation occurred at 9.5 years old. The same hormone profile was observed with primary adrenal insufficiency (Table 2, because of oral contraceptive medication, only a few hormones were dosed).

This hormone profile was not consistent with the diagnostic of the most common enzymatic blocks, as no elevation of any single steroid was observed. However, this was suggesting an enzymatic deficiency at a very early step in steroid biosynthesis. As the patient's adrenals did not appear enlarged on computed tomography, STAR deficiency was highly improbable. The hypothesis of P450Scc deficiency was conducted by *CYP11A1* gene analysis by next-generation sequencing (NM_000781 [8]). Both patients were then found to be compound heterozygous for the mutations 940G > A (p.Glu314Lys) and c.1393C > T (p.Arg465Trp), and haplotype segregation was confirmed by parental genetic analysis. The past medical history of the father was marked by surgery for hypospadias. In addition, to testosterone replacement for the male patient, treatment consisted in glucocorticoid substitution without the need for mineralocorticoids, as blood pressure, natremia, and kaliemia were normal in both patients.

## 3. Discussion

TART frequency in enzymatic blocks is estimated between 14 and 86% according to a publication gathering 33 studies in this field, with underestimation probably due to undone screenings [7]. These lesions should be actually clinically palpable if the size is more than 20 mm and are bilateral in almost 80% of cases [7]. Studies show that TARTs can be present from childhood, with a higher prevalence during puberty [3]. Their prevalence could be correlated with ACTH levels in patients treated with hydrocortisone, as suggested in a cohort study of 25 patients where the increasing dose of hydrocortisone led to a size reduction of lesions in 35.7% of cases and a remission in 42.9% at 6 month follow-up [9]. These lesions are thus reversible. In summary, TARTs were reported mainly in CAHs with bad hormone control, but it is to know that TARTs can also develop in well-controlled patients, and conversely, bad-controlled patients can be free from TART occurrence [7].

The TART origin could be due to the development of embryonic primordial adrenogenital cells or from urogenital cell ridges [10]. These tumors could be made of fœtal Leydig cells or precursors of Leydig cells [10]. These lesions are usually located in the mediastinum, testis or the testicular hilus [3, 7]. If TARTs are benign, they can compress the seminiferous tubules and lead to obstructive azoospermia with peritubular fibrosis and irreversible damage to the peritesticular tissue [1, 10]. This can lead to infertility or endocrine defects [3, 10]. A study of 30 CAH, mainly defects in 21-hydroxylase, reported an 86% TART, responsible for spermogram abnormalities in 43% of cases [11].

A deleterious paracrine effect is also suspected for Leydig cells or neighbour germinal cells, due to a potentially toxic effect of local steroids production [1]. Some blocks can finally lead to fertility troubles due to both tumoral invasion and lipid accumulation within steroidogenic cells [4].

TART lesions are mostly echographycally irregular, hypoechogenic, and homogenous, but can also be heterogeneous, or with a round shape, and rarely iso or hyperechogenic [3]. They can contain hyperechogenic elements due to fibrosis or calcifications, especially in adults [3]. In a study recording individuals younger than 18 years old, the size of the lesions was measured between 0.2 and 3.8 cm [3]. TARTs are classically highly vascularized [3, 7]. In MRI, these lesions are T1 hyper or isosignal, and T2 hyposignal, with a clean edge [7]. Imaging follow-up is of high importance as the aspects of ultrasonography may vary over time [12].

These lesions are macroscopically firm, yellow to brown-coloured, round, oval or multilobular [7]. It can be difficult to distinguish TART from mature Leydig cell tumors (LCT), which are the most common stromal testicular neoplasms [1]. It is important to distinguish between these tumors because the treatment is different, whereas the treatment of TART is mainly medical with the prescription of oral drugs, LCT management is surgical. Indeed, TARTs should not be operated except in cases of pain or discomfort due to their high volume [7]. Studies gather interesting histological and genetic markers that can help distinguish one from the other [7].

The patients reported in our paper harbor an extremely rare genetic autosomal recessive disorder with around 40 described families and 30 different mutations [4, 13–17]. There is actually a wide spectrum of different clinical forms within P450Scc defects. In classic form, salt wasting is observed with female genitals for the 46XY subjects. Partial defects are associated with the late onset of adrenal insufficiency or cortisol insufficiency with normal external genitalia, or with various degrees of underandrogenisation. The first description of the late onset of adrenal insufficiency in P450Scc deficiency was published in 2009 [13], where a boy with cryptorchidy and hypospadias presented adrenal insufficiency at 9 years of age. Mild forms were then reported in several families. Those forms seem to be explained by the residual activity of the mutated enzyme, compared to wild-type enzymes according to functional studies. P450Scc deficiency is often grouped with STAR (steroidogenic acute regulatory protein) deficiency under the term of congenial lipoid adrenal hyperplasia (CLAH). STAR allows cholesterol import into the mitochondrial membrane. However, the comparison between P450Scc and STAR deficiencies is controversial as enlarged adrenal glands are not reported in P450Scc deficiency [4].

As far as our patients' mutations are concerned, Goursaud et al. have reported that the functional consequences of these mutations can explain an uncomplete phenotype [8]. Both mutations, c.940G > A or p.Glu314Lys and c.1393C > T or p.Arg465Trp, were found in two families in a compound heterozygous state [8]. Goursaud et al. provided biological proof that p.Arg465Trp completely abolished CYP11A1 activity. Conversely, c.940G > A or p.Glu314Lys seems responsible for the mild phenotype due to its partial splicing impact and a mutated protein that maintains its activity [8]. Maharaj published in 2019 the report of 13 additional families with the p.Glu314Lys mutation and a second rare disruptive or synonymous mutation variant in *CYP11A1*, suggesting that mutations of the gene coding for P450Scc enzyme should be more frequent than previously thought [18].

The diagnosis of the delayed onset of the P450Scc mutation is difficult. Kallali et al. published the case of 3 brothers, among whom one had unilateral orchiectomy at 25 years of age, with the initial conclusion of Leydig cell hyperplasia [4]. The 3 brothers had glucocorticoid deficiency and partial deficiency in mineralocorticoids [4]. Other cases of orchiectomies were reported in patients with TART, leading to the need for endocrinologists to better communicate with urologists to avoid unnecessary surgeries [19, 20].

This observation illustrates the difficulty to diagnose primary adrenal insufficiency in adults, especially with mild phenotypes. On one hand, when a block is already known in an individual presenting CAH, the diagnosis of TART is commonly and easily made with ultrasonography, with no need for biopsy or partial orchiectomy that should indeed be avoided. On the other hand, the new discovery of bilateral testicular lesions in an adult should evoke a TART and lead to minimal endocrinological exploration. The evolution of unoperated TART is classically a regression under medical glucocorticoid treatment.

## Figures and Tables

**Table 1:** Hormone profiles of the 31-year-old male patient harboring compound heterozygous mutations in the *CYP11A1* gene.

Hormone	Unit	Result	Normal range
Cortisol	nmol/L	72	110.7–609.2
ACTH	pg/ml	>1250	10–60
Total testosterone	nmol/L	7.9	6–27
Free testosterone	pmolL	14.2	28.8–139
Estradiol	pmol/L	92	33–228
SHBG	nmol/L	48.83	14.5–48.4
FSH	UI/L	74	1.3–9.2
LH	UI/L	31.9	1.2–8.6
Delta 4 androstenedione	ng/ml	0.24	0.5–3.5
Dehydroepiandrosterone sulfate	ng/ml	497	1600–4490
Progesterone	nmol/L	0.8	<0.64
17-Hydroxyprogesterone	nmol/L	0.79	1.81–6.6
Pregnenolone	nmol/L	0.32	0.6–1.9
17-Hydroxypregnenolone	nmol/L	0.6	1.8–10.2
11-Deoxycortisol	nmol/L	0.29	0.6–3.2
11-Deoxycorticosterone	pmol/L	191	121–514
Corticosterone	nmol/L	5.8	5.7–23
Aldosterone	pmo/L	180	117–580
Active renin	mUI/L	18.54	<36

**Table 2:** Hormone profile of the 29-year-old female patient harboring compound heterozygous mutations in the *CYP11A1* gene.

Hormone	Unit	Result	Normal range
Cortisol	nmol/L	226	186.1–664.8
ACTH	pmol/L	>440	1.1–13.2
Testosterone	nmol/L	0.14	<3.3
Delta 4 androstenedione	nmol/L	0.7	1.4–9.78
Dehydroepiandrosterone sulfate	nmol/L	1437	2680–9230
Aldosterone	pmol/L	294	117–580
Active renin	mUI/L	12.8	<36

## Data Availability

The data used to support the findings of this work are included within the article.
